# The Metabolic Pathway of Bile Secretion Is Vulnerable to *Salmonella enterica* Exposure in Porcine Intestinal Epithelial Cells

**DOI:** 10.3390/ani14050789

**Published:** 2024-03-03

**Authors:** Jiajia Cai, Xiaolei Chen, Chao Xu, Xiaoyang Zhu, Haifei Wang, Shenglong Wu, Demin Cai, Hairui Fan

**Affiliations:** 1 Joint International Research Laboratory of Agriculture & Agri-Product Safety, The Ministry of Education of China, Yangzhou University, Yangzhou 225009, China; jjcai@yzu.edu.cn (J.C.); slwu@yzu.edu.cn (S.W.); 2 Key Laboratory for Animal Genetics, Breeding, Reproduction and Molecular Design, College of Animal Science and Technology, Yangzhou University, Yangzhou 225009, China; mx120230917@stu.yzu.edu.cn (X.C.); mx120200807@stu.yzu.edu.cn (C.X.); mz120211472@stu.yzu.edu.cn (X.Z.); hyfiwang@yzu.edu.cn (H.W.); 3 International Joint Research Laboratory in Universities of Jiangsu Province of China for Domestic Animal Germplasm Resources and Genetic Improvement, Yangzhou University, Yangzhou 225009, China; 4 College of Animal Science and Technology, Yangzhou University, Yangzhou 225009, China

**Keywords:** *Salmonella enterica*, pigs, transcriptome, IPEC-J2, bile, pathway signals

## Abstract

**Simple Summary:**

*Salmonella enterica* is a zoonotic pathogen in humans that causes gastrointestinal infections or more serious infections. Second only to poultry, pigs are the most common source of zoonotic *Salmonella enterica* infections. Although current pig farm disinfection is very strict, its positive detection rate is still very high. Resistance to commonly used antibiotics has been observed in various bacteria in recent decades, requiring the use of alternative strategies to control pathogens. Therefore, comprehension of the pathogenic mechanism of *Salmonella enterica* in the pig gut is imperative, as it will greatly facilitate the advancement of preventive and therapeutic approaches for combating *Salmonella enterica*. By exploring the transcriptional regulation of intestinal cells in response to *Salmonella enterica* infection, we can find the key genes and pathways of host cells in resistance to *Salmonella enterica* infection, which will provide theoretical support for disease resistance breeding in pigs.

**Abstract:**

Pigs can be colonized with *Salmonella enterica* and become established carriers. However, the mechanisms of the host’s response to *Salmonella enterica* infection are largely unclear. This study was constructed with the *Salmonella enterica* infection model in vitro using porcine intestinal epithelial cells (IPEC-J2). Transcriptome profiling of IPEC-J2 cells was carried out to characterize the effect of *Salmonella enterica* infection and lipopolysaccharide (LPS) treatment, in which LPS-induced inflammation was a positive control. At first, *Salmonella enterica* infection increased the cell apoptosis rate and induced an inflammation response in IPEC-J2. Then, the up-regulated genes were enriched in metabolic pathways, such as those for bile secretion and mineral absorption, while down-regulated genes were enriched in immune-related pathways, such as the Toll-like receptor signaling and p53 signaling pathways. Moreover, we found 368 up-regulated genes and 101 down-regulated genes in common. Then, an integrative analysis of the transcriptomic profile under *Salmonella enterica* infection and LPS treatment was conducted, and eight up-regulated genes and one down-regulated gene were detected. Among them, *AQP8* is one critical gene of the bile secretion pathway, and its mRNA and protein expression were increased significantly under *Salmonella enterica* infection and LPS treatment. Thus, the AQP8 gene and bile secretion pathway may be important in IPEC-J2 cells under *Salmonella enterica* infection or LPS treatment.

## 1. Introduction

The *Salmonella* bacterium, a prevalent foodborne pathogen, exhibits extensive distribution across global pork production chains and traverses the various stages of livestock and poultry processing [1,2]. The transmission of *Salmonella enterica* from animals to humans typically occurs through the consumption of animal-derived food that has been contaminated, as well as through direct or indirect contact with animal excrement [3]. In certain individuals, the infection disseminates by invading the intestinal epithelium and internalizing within phagocytes, leading to subsequent dissemination [4]. The zoonotic pathogen *Salmonella enterica*, which causes humans to develop gastrointestinal infections or more severe ailments, ranks as the second most prevalent zoonotic organism reported in the European Union, following closely behind *Campylobacter* spp. [5]. Although poultry and eggs are widely acknowledged as the predominant source of zoonotic *Salmonella* infection in Europe, pigs emerge as the second most prevalent reservoir [5]. In total, 239 *Salmonella* strains were isolated from 1389 random samples obtained from a single pig farm in China, with a positive rate of 17.2%. Most of the strains were detected in fecal samples (26.3%) and feed samples (7.1%) [1]. The control and limitation of *Salmonella enterica* on pig farms are currently considered important in reducing the risk of zoonotic transmission [3]. The incidence of *Salmonella enterica* infection among weaned and finishing pigs is alarmingly high, and salmonellosis in swine can lead to diminished weight gain and even mortality [6]. However, *Salmonella enterica* infection in pigs is usually subclinical, and poses challenges in the identification of infected pigs, implementation of targeted controls, reduction in transmission risks within a farm setting, and prevention of entry into *Salmonella*-negative herds [3]. Meanwhile, over the past few decades, various bacteria have developed resistance to commonly used antibiotics, necessitating the implementation of alternative strategies for pathogen control [7,8,9]. Therefore, comprehension of the pathogenic mechanism of *Salmonella enterica* in the pig gut is imperative, as it will greatly facilitate the advancement of preventive and therapeutic approaches for combating *Salmonella enterica*. By exploring the transcriptional regulation of intestinal cells in response to *Salmonella enterica* infection, we can find the key genes and pathways of host cells in resistance to *Salmonella enterica* infection, which will provide theoretical support for disease resistance breeding in pigs.

*Salmonella* chromosomes contain a variety of virulence mechanisms to accomplish the pathogenic process, and the most crucial virulence genes are those situated within the renowned *Salmonella* pathogenicity islands (SPIs), which have been reported to significantly contribute to its pathogenesis [4]. The genes *hilA* and *invA* are the main virulence genes of the SPIs; among them, *hilA* is indispensable to *Salmonella* for the invasion of epithelial cells and downstream gene transcription regulation [4,10]. The *invA* gene of the *Salmonella* species empowers the bacteria to infiltrate the host and initiate infection, thereby enhancing the level of pathogenicity exhibited by the isolates [4]. The expression of *hilA* and *invA* has been used to as way to identify the invasive serovars of *Salmonella enterica* [11]. Moreover, lipopolysaccharides (LPS), which induce the pathogenic factor in Gram-negative bacteria, could activate innate immunity and lead to injury [12,13], and are usually used to build a cellular inflammation model in vitro for research on the regulation of key genes and pathways in a variety of cells [14,15,16]. For better research on the transcriptional regulation of porcine gut cells in response to *Salmonella enterica* infection, we also constructed an LPS-induced inflammation model as a reference.

In this study, we constructed the *Salmonella enterica* infection model in vitro using porcine intestinal epithelial cells (IPEC-J2). The transcriptome profile of IPEC-J2 cells was characterized under *Salmonella enterica* infection and LPS treatment. Bioinformatics analysis was performed and revealed alterations at the transcription level and in the enriched biological signaling pathways of IPEC-J2 cells after in vitro *Salmonella enterica* infection and LPS treatment. Moreover, the signature genes and enriched pathways were identified. Our study will provide a theoretical basis and putative pathway for resisting *Salmonella enterica* infection and resistance breeding in pigs and other animals.

## 2. Materials and Methods

### 2.1. Bacterial Culture

*Salmonella enterica* (BNCC186354, BNCC; Xinyang, China) bacteria were incubated with LB culture medium overnight at 200 r/min on a rocking platform, and then collected at 4000 rpm for 5 min and washed three times with phosphate-buffered saline (PBS) (Gibco, Grand Island, NY, USA).

### 2.2. Salmonella enterica Strain Infection

**Figure 1:**
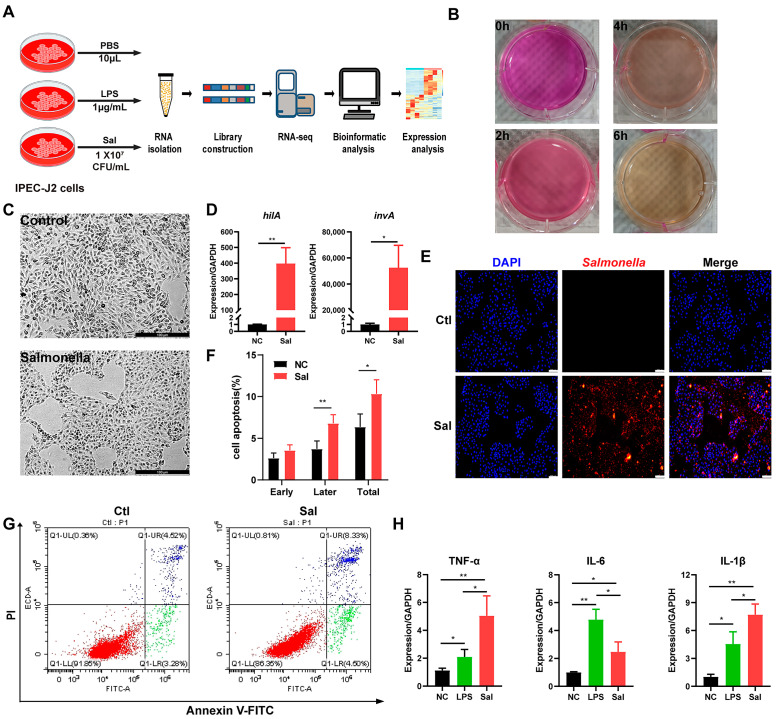
Evaluation of the deleterious effect of *Salmonella enterica* on IPEC-J2 cells. (**A**) Outline of the experiment and data analysis workflow. (**B**) Alteration in the medium under *Salmonella* infection for different periods of time (2, 4 and 6 h). (**C**) Morphology analysis of IPEC-J2 cells under *Salmonella enterica* infection for 4 h. Scale bars are 100 μm. (**D**) Expression of *hilA* and *invA* of IPEC-J2 cells under *Salmonella enterica* infection for 4 h (*n* = 3). (**E**) Representative image of *Salmonella* (red) and nuclei (blue) in infected cells. Scale bars are 100 μm. (**F**) Apoptosis of IPEC-J2 cells under *Salmonella enterica* evaluated via the measurement of Annexin-V and PI using flow cytometry (*n* = 3). (**G**) The figure displays representative staining, with quadrant numbers indicating subpopulation percentages. The later apoptosis cells are shown as Annexin V-positive and PI-positive (bule), and the early apoptosis cells are shown as Annexin V-positive and PI-negative (green), and the normal cells are shown as Annexin V-negative and PI-negative (red). (**H**) The expression of *TNF-α*, *IL-6*, and *IL-1β* of IPEC-J2 cells under *Salmonella enterica* infection and LPS treatment (*n* = 3). Sal: *Salmonella enterica*; Ctl: control group; LPS: lipopolysaccharide group. *, *p* < 0.05; **, *p* < 0.01 (*t*-test).

IPEC-J2 cells were seeded into 6-well cell plates (Corning Inc., Corning, NY, USA) at a concentration of 1 × 10^6^ cells/mL. The cells were cultured in Dulbecco’s modified Eagle medium (DMEM) containing 10% fetal bovine serum (FBS, Gibco, Grand Island, NY, USA) in a cell incubator (37 °C, 5% CO_2_). After the cells grew to reach 80% confluency, the cells were incubated for 4 h with *Salmonella enterica* (1 × 10^7^ CFU/mL) [17] or LPS (1 µg/mL), without antibiotics [18]. For the control group, a comparable amount of PBS was replaced, and then gentamicin (10 μg/ml) was added to the media to kill extracellular bacteria [19]. The workflow of this experiment is described in Figure 1A.

### 2.3. Detection of mRNA Expression of hilA and invA

The cells and adherent bacteria were collected, and total RNAs were extracted using Trizol (Takara, Dalian, China). Then, PrimerScript RT Reagent Kit (Vazyme Biotech Co., Ltd., Nanjing, China) was used for the reverse transcription of the RNA. The mRNA expression of *hilA* and *invA* were detected via RT-qPCR (Q511-02, Vazyme Biotech Co., Ltd., Nanjing, China) [17]. For normalization, 16S rRNA was used as the housekeeping gene. The primer details are listed in Table S1.

### 2.4. Cell Apoptosis Assay

Cell apoptosis assays were conducted in accordance with the instructions of Apoptosis Analysis Kit (Solarbio, Beijing, China). Each measurement was conducted three times utilizing no less than 10,000 cells.

### 2.5. Immunofluorescence Microscopy

Adherence detection of *Salmonella enterica* in vitro was conducted as described previously [19]. Briefly, the IPEC-J2 cells were seeded on a 12-well plate (Corning Inc., Corning, NY, USA) with coverslips at a concertation of 5 × 10^5^ cells/mL for 12 h. Then, 1 × 10^9^ CFU of *Salmonella enterica* was added to the cell media for 4 h at 37 °C. PBS containing 4% PFA was used to fix the treated cells, and then the treated cells were blocked in PBS with 3% BSA and 0.2% TritonX-100, and incubated with anti-*Salmonella* (1:20, ab35156, Abcam, Cambridge, UK) for 12 h. Alexa Fluor 555-conjugated antibody was used to detect the signal. DNA was stained with 6 μg/ml of DAPI for 3 min. The coverslips were then reverse-buckled onto the slide and sealed using a fluorescence quencher (Vector Laboratories, Newark, CA, USA). Images were captured by using a Leica fluorescence microscope (Leica, Wetzlar, Germany).

### 2.6. RNA-Seq Data Processing

The treated IPEC-J2 cells were collected for RNA-seq, including those in the control group (Ctl, PBS, *n* = 4), *Salmonella enterica* group (Sal, *n* = 4), and LPS (L2880, Sigma-Aldrich, St. Louis, MO, USA, *n* = 4) group.

The in-house Perl scripts were performed to process all raw reads. Briefly, Trimmomatic was used to remove the adapter and trim low-quality bases, and to calculate the Q20, Q30, and GC contents. The reference genome index (Sscrofa11.1) was built, and the Hisat2 (version 2.0.5) was used to align it to paired-end clean reads. Htseq-count was conducted to count the number of reads, which mapped to each gene; then, the FPKM of each gene was calculated based on its length and read counts. The DESeq2 R package (1.16.1) was used to analyze differentially expressing genes (DEGs) with an adjusted *p*-value < 0.05 and a|log2 fold change| ≥ 1.2.

### 2.7. Gene Ontology and Pathway Analysis

In this study, gene ontology (GO) analysis was conducted to elucidate the biological implications of the differentially expressed gene sets of the different treated groups. Meanwhile, pathway analysis was performed to explore the enriched signaling pathways of the differentially expressed gene sets, based on the Kyoto Encyclopedia of Genes and Genomes (KEGG) database. Then, the significant pathway and GO categories were identified using Fisher’s exact test with a *p*-value < 0.05 as the cut-off [20].

### 2.8. Quantitative Real-Time Polymerase Chain Reaction (RT-qPCR)

Total RNAs of different treated IPEC-J2 cells were extracted with RNAiso (Takara, Dalian, China). Next, the 1 µg RNA was reversely transcribed into complementary DNA (cDNA) using a cDNA synthesis kit (Vazyme Biotech Co., Ltd., Nanjing, China). The mRNA expression of DGEs was detected via RT-qPCR (Q511-02, Vazyme Biotech Co., Ltd., Nanjing, China). The expression of *GAPDH* was used for normalizing the other gene levels. The 2^−∆∆Ct^ method was used to calculate relative gene expression [21]. The primer details are listed in Table S1.

### 2.9. Western Blotting

Total proteins of differently treated IPEC-J2 cells were extracted with RIPA buffer (Applygen, Beijing, China) supplemented with protease inhibitors, incubated on ice for 20 mins, and then centrifuged at 4 °C for 15 min at 12,000 rpm. The BCA protein assay kit (Solarbio, Beijing, China) was used to quantify the amount of protein; after the lysated proteins were denatured at 98 °C for 10 min, equal amounts (20 μg) of proteins each group were loaded on 10% SDS-PAGE gel, and after gel electrophoresis, the proteins were transferred onto polyvinylidene difluoride (PVDF) membranes (Immobilon, Darmstadt, Germany) for 1 h. Next, the proteins were blocked with 5% skimmed milk at room temperature for 1 h, and washed with 1× TBST (TBS with Tween-20, Solarbio, Beijing, China) three times. The blocked membranes were incubated with primary antibodies (Anti-AQP8, Anti-P53, anti-PCNA, anti-Occludin, and anti-HSP90) overnight at 4 °C, and then incubated with secondary antibodies (1:5000) at room temperature for 2 h. After being washed with 1× TBST, and the PVDF membranes were incubated in Luminol/Enhancer Reagent (New Cell & Molecular Biotech, Suzhou, China) for 1 min. Finally, the relative intensities were analyzed using the FluorChem FC3 system (Protein-Simple, San Jose, CA, USA). Anti-P53 (ab26), anti-PCNA (ab29), and anti-Occludin (ab222691) antibodies were purchased from Abcam Ltd. (Cambridge, UK). Anti-AQP8 (sc-81870) was purchased from Santa Cruz Biotechnology, Inc. (Dallas, TX, USA). The anti-HSP90 (60318) antibody was obtained from Proteintech Ltd. (Rosemont, IL, USA).

### 2.10. Statistical Analysis

All results are expressed as the mean ± SD from three separate experiments. An unpaired Student *t*-test was used to compare different groups. GraphPad Prism 8.0.1 software (GraphPad, La Jolla, CA, USA) was used to generate figures, with the significance level set to 0.05.

## 3. Results

### 3.1. Evaluation of the Deleterious Effect of Salmonella enterica on IPEC-J2 Cells

In order to investigate the deleterious effect of *Salmonella enterica*, the IPEC-J2 cells were infected with *Salmonella enterica* for 2 h, 4 h, and 6 h. The results showed that *Salmonella enterica* proliferated continuously, and the color of the medium deepened with time (Figure 1B). Morphology analysis of IPEC-J2 cells infected with *Salmonella enterica* for 4 h was performed, and we found cell shrinkage, a loss of the spindle shape, and cell shedding (Figure 1C). The analysis of the mRNA expression levels of *hilA* and *invA* indicated that *Salmonella enterica* significantly increased the expression of these two genes (Figure 1D). The IHC results also demonstrate that *Salmonella enterica* (red) adhered to the IPEC-J2 cells (Figure 1E). In addition, the apoptosis of IPEC-J2 cells was significantly enhanced (Figure 1F,G). To further verify the deleterious effect of *Salmonella enterica* on IPEC-J2, the IPEC-J2 cells were exposed to LPS (1 μg/mL) for 4 h, and then the mRNA expression levels of inflammatory factors (*TNF-α*, *IL-6*, and *IL-1β*) were detected. The results showed that *Salmonella enterica* and LPS caused an inflammatory response in IPEC-J2 cells (Figure 1E).

### 3.2. Alterations in the Transcriptomic and Biological Processes in IPEC-J2 Cells under Salmonella enterica Infection

**Figure 2:**
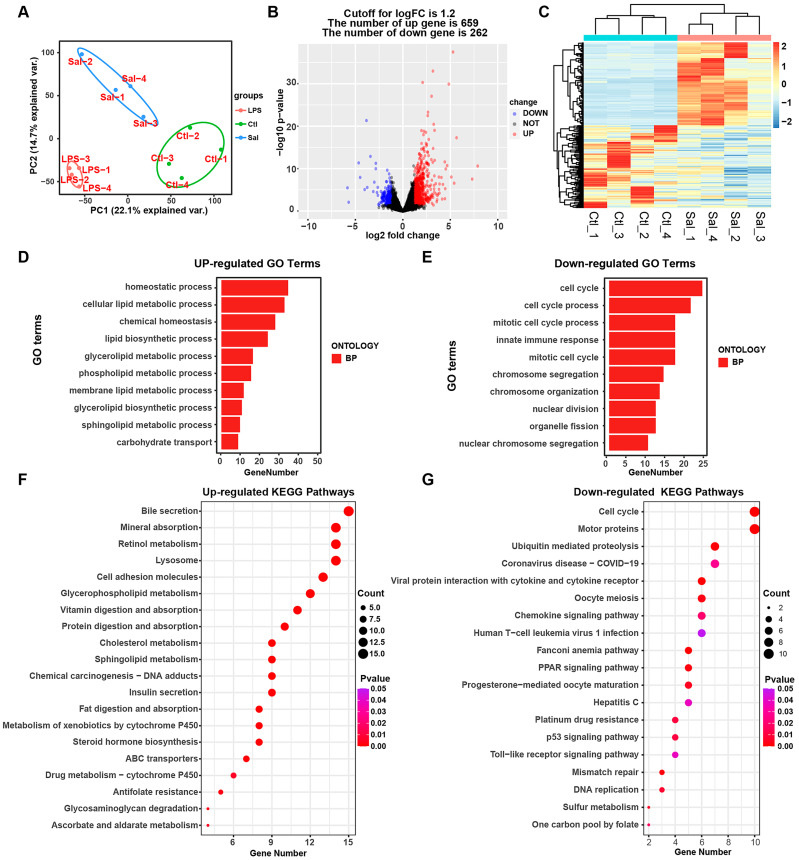
Analysis of transcriptome profiles between the *Salmonella enterica*-infected and control groups. (**A**) Principal component analysis (PCA) of all the RNA-seq samples. (**B**) Volcano plot and heatmap (**C**) of differential expression profiles. (**D**,**E**) Top 10 enriched GO terms of up-regulated and down-regulated genes. (**F**,**G**) Top 20 enriched KEGG pathway terms of up-regulated and down-regulated genes. Sal: *Salmonella enterica*; Ctl: control group.

For the clarification of the pathogenic mechanism of *Salmonella enterica* in IPEC-J2 cells, we analyzed the characterization of the transcriptomic alterations in the IPEC-J2 cells in response to *Salmonella enterica* infection. For each group, we obtained four independent RNA-seq replicates, and PCA analysis indicated that all the samples were clustered by group, as depicted in Figure 2A. These results show that our data could effectively reflect the genome-wide gene expression profile of the tested sample. A volcano plot of DEGs from the RNA-seq data is shown in Figure 2B, and the up-regulated genes (659) and down-regulated genes (262) with high variability in IPEC-J2 cells upon *Salmonella enterica* exposure are listed in Figure 2C and Table S2. In addition, after the GO terms were analyzed, it was found that the homeostatic process, the cellular lipid metabolic process, and chemical homeostasis were induced in *Salmonella enterica*-associated biological processes, while the cell cycle, cell cycle process, mitotic cell cycle process, and innate immune response were reduced in *Salmonella enterica*-associated biological processes (Figure 2D,E, Table S3). Moreover, the up-regulated differential genes were enriched in metabolic pathways, such as those for bile secretion, mineral absorption, retinol metabolism, etc. Meanwhile, the down-regulated differential genes were enriched in the pathways for the cell cycle, motor proteins, ubiquitin-mediated proteolysis, etc. Among them, the human T-cell leukemia virus 1 infection and Toll-like receptor signaling pathways were enriched significantly (Figure 2F,G, Table S4). These enrichments of the pathways and biological processes indicate that the IPEC-J2 cell response to *Salmonella enterica* infection is an intricate process.

### 3.3. Alterations in the Transcriptomic and Biological Processes in IPEC-J2 Cells upon LPS Treatment

**Figure 3:**
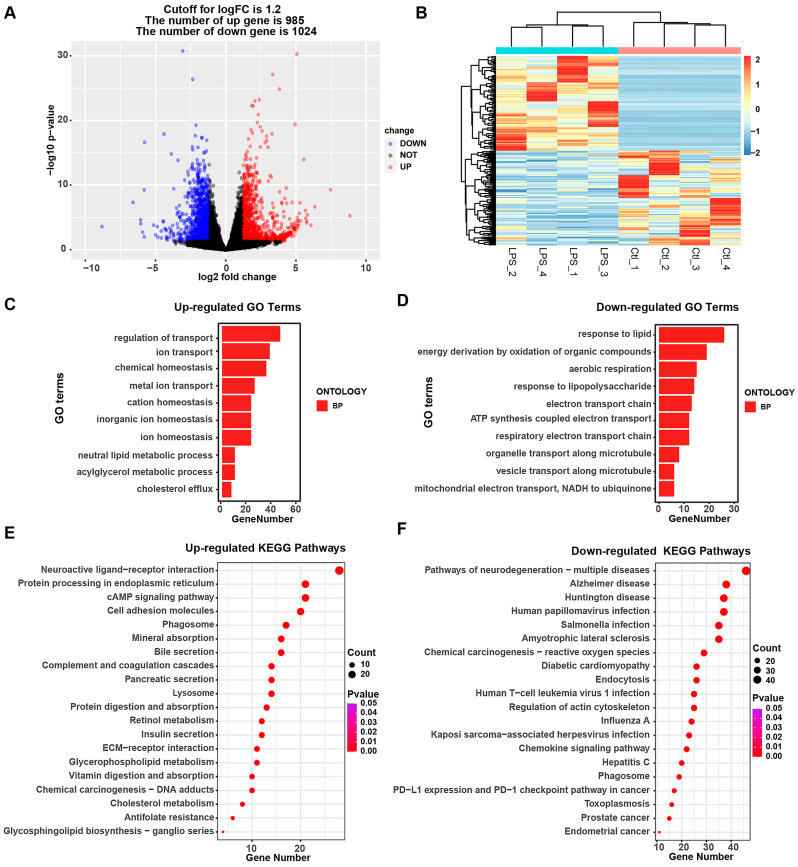
Analysis of transcriptome profiles between the LPS-treated and control groups. (**A**) Volcano plot and heatmap (**B**) of differential expression profiles. (**C**,**D**) Top 10 enriched GO terms of up-regulated and down-regulated genes. (**E**,**F**) Top 20 enriched KEGG pathway terms of up-regulated and down-regulated genes. LPS: lipopolysaccharide group; Ctl: control group.

For further clarification of the pathogenic mechanism of *Salmonella enterica* in IPEC-J2 cells, we also analyzed the characterization of the transcriptomic alterations in IPEC-J2 cells in response to LPS treatment. After analysis, 985 up-regulated genes and 1024 down-regulated genes were detected (Figure 3A,B, Table S5). The alteration was larger than that under *Salmonella enterica* infection. Meanwhile, the GO terms were analyzed, and it was found that the regulation of transport, ion transport, chemical homeostasis, etc., was induced in LPS-associated biological processes (Figure 3C). Meanwhile, the response to lipids, energy derivation due to the oxidation of organic compounds, aerobic respiration, the response to lipopolysaccharides, etc., were reduced in LPS-associated biological processes (Figure 3D, Table S6). In addition, KEGG pathway analysis was also performed, and we found that the up-regulated differential genes were enriched in neuroactive ligand–receptor interactions, protein processing in the endoplasmic reticulum, the cAMP signaling pathway, the PI3K-Akt signaling pathway, etc. Among them, the bile secretion, mineral absorption, and insulin secretion pathways had similar responses to *Salmonella enterica* (Figure 3E, Table S7), while the down-regulated differential genes were enriched in multiple neurodegeneration diseases, including Alzheimer’s disease, Huntington’s disease, etc. (Figure 3F, Table S7).

### 3.4. Integrative Analysis of the Transcriptomic Profile of IPEC-J2 Cells under Salmonella enterica and LPS Exposure

**Figure 4:**
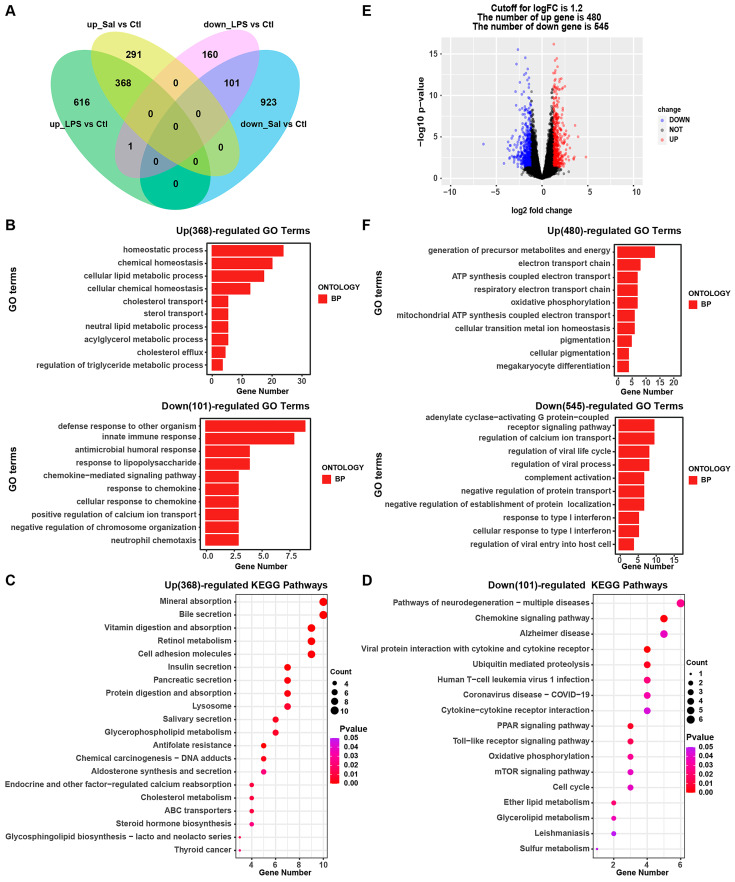
Integrative analysis of the transcriptomic profile of IPEC-J2 cells under *Salmonella enterica* and LPS exposure. (**A**) Venn diagram showing common DEGs of IPEC-J2 cells under *Salmonella enterica* and LPS exposure compared with those of the control group. (**B**) Top 10 enriched GO terms of up- and down-regulated genes. (**C**,**D**) Top 20 enriched KEGG pathway terms of up- and down-regulated genes. (**E**) Volcano plot of differential expression profiles of IPEC-J2 cells under Sal vs. LPS exposure. (**F**) Top 10 enriched GO terms of IPEC-J2 cells under Sal vs. LPS exposure with up- and down-regulated genes. Sal: *Salmonella enterica*; Ctl: control group; LPS: lipopolysaccharide group.

To further understand the mechanism of inflammation caused by *Salmonella enterica*, we performed an integrative analysis of the transcriptomic profile of IPEC-J2 cells under *Salmonella enterica* and LPS exposure. We compared the up- and down-regulated genes between the Sal and Ctl groups and between the LPS and Ctl groups, and we found 368 up-regulated genes and 101 down-regulated genes (Figure 4A, Table S8). These results indicate that the responses of IPEC-J2 cells after Sal and LPS exposure are largely similar. Meanwhile, GO and KEGG analyses were performed with these DEGs (368 up-regulated genes and 101 down-regulated genes), and we found that the homeostatic process, chemical homeostasis, the cellular lipid metabolic process, etc., were induced in LPS and *Salmonella enterica* biological processes, while the defense response to other organisms, innate immune response, antimicrobial humoral response, response to lipopolysaccharides, etc., were reduced in LPS- and *Salmonella enterica*-associated biological processes (Figure 4B, Table S9). In addition, these up-regulated differential genes were enriched in the mineral absorption, bile secretion, and vitamin digestion and absorption pathways, among others (Figure 4C), while the down-regulated genes were enriched in pathways of neurodegeneration including multiple diseases such as Alzheimer’s disease, the chemokine signaling pathway, Toll-like receptor signaling pathway, etc. (Figure 4D, Table S10).

Meanwhile, we compared the difference in the transcriptome profile of IPEC-J2 cells exposed to *Salmonella enterica* and LPS. In total, 480 up-regulated genes and 545 down-regulated genes were detected (Figure 4E, Table S11). GO and KEGG analyses were performed, and it was found that the generation of precursor metabolites and energy, electron transport chains, and ATP synthesis coupled with electron transport were induced, while adenylate cyclase-activating G protein-coupled receptor signaling, the regulation of calcium ion transport, and the regulation of the viral life cycle were reduced (Figure 4F, Table S12). The up-regulated differential genes were enriched due to *Salmonella enterica* infection, chemical carcinogenesis-reactive oxygen species, lipids, and atherosclerosis (Figure S1A), while the down-regulated differential genes were enriched due to neuroactive ligand–receptor interactions, the cAMP signaling pathway, human papillomavirus1 infection, etc. (Figure S1B, Table S13). The findings suggest that there exist disparities in the reactions of IPEC-J2 cells when exposed to Sal and LPS.

### 3.5. AQP8 (Aquaporin 8) Gene and Bile Secretion Pathway May Be Newly Important in IPEC-J2 Cells under Salmonella enterica or LPS Exposure

**Figure 5:**
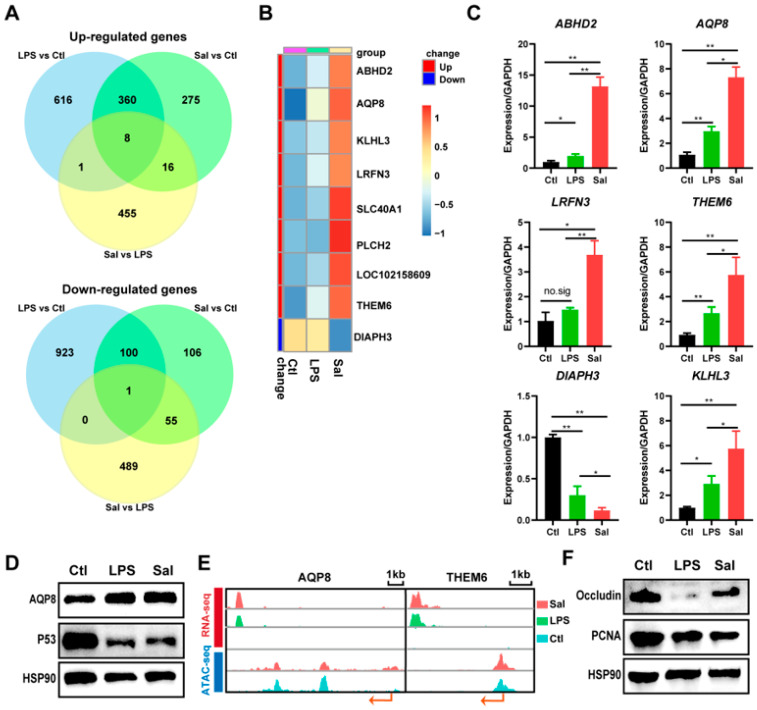
Relative expression levels of the DGEs. (**A**) Venn diagram showing the DEGs of IPEC-J2 cells under *Salmonella enterica* and LPS exposure compared with those of the control group. (**B**) Heatmap of 8 up-regulated genes and 1 down-regulated gene of IPEC-J2 cells under *Salmonella enterica* infection vs. LPS treatment. (**C**) Relative mRNA expression levels of the DGEs. * *p* < 0.05; ** *p* < 0.01. (**D**) Western blot analysis of the relative expressions of AQP8 and P53, which is the key protein of the p53 signaling pathway. (**E**) Visualization of the expression and chromatin accessibility of the *AQP8* and *THEM6* genes. Arrows indicate the position and direction of transcription initiation. (**F**) Western blot analysis of the relative expression of Occludin and PCNA. Sal: *Salmonella enterica*; Ctl: control group; LPS: lipopolysaccharide group.

To validate the data on RNA-seq, we further analyzed the DGEs from the Sal, LPS, and control groups; eight up-regulated genes and one down-regulated gene were detected (Figure 5A). These included *ABHD2*, *AQP8*, *KLHI3*, *LOC102158609*, *LRFN3*, *PLCH2*, *SLC40A1*, *THEM6,* and *DIAPH3* (Figure 5B). Among them, *ABHD2*, *AQP8*, *KLHI3*, *LRFN3*, *THEM6*, and *DIAPH3* were quantified via qRT-PCR, and the results indicated that these DEGs exhibit similar expression patterns to those identified in the RNA-seq analyses (Figure 5B,C). The protein expression level analysis showed that the expression level of AQP8 was enhanced under *Salmonella enterica* infection and LPS treatment (Figure 5D). For further clarification of the alterations in these DGEs, we used the previously reported ATAC-seq data from IPEC-J2 cells under *Salmonella enterica* infection [17], and as randomly displayed genes, *AQP8* and *THEM6* are shown in Figure 5E to have different expression and peak enrichment values in genomics, as determined using an integrative genomics viewer (IGV). The protein expression levels of P53, a key protein in the P53 signaling pathway, and PCNA, a key protein in the cell cycle pathway, were significantly decreased after *Salmonella enterica* and LPS exposure in IPEC-J2 cells. Furthermore, the protein expression levels of tight junction proteins including Occludin were detected and showed a significant decrease after *Salmonella enterica* and LPS exposure. Furthermore, the protein expression levels of Occludin, a crucial component of tight junctions, were analyzed and exhibited a significant decrease following *Salmonella enterica* and LPS exposure in IPEC-J2 cells. *AQP8* is the one gene of the bile secretion pathway, and this pathway is on top within the *Salmonella enterica* and LPS-treated groups compared with that in the control group. Thus, the *AQP8* gene may be a new important gene, the bile secretion pathway may be a new important pathway in IPEC-J2 cells under *Salmonella enterica* or LPS exposure.

## 4. Discussion

The primary enteric pathogen *Salmonella enterica* is capable of infecting both humans and animals, initiating an infection upon the consumption of contaminated food or water, allowing *Salmonella* to reach the intestinal epithelium and cause gastrointestinal disease [4]. It is reported that seven different serotypes of *Salmonella* were identified on one pig farm in China, including *Salmonella Derby* (21.8%), *Salmonella Typhimurium* (18.8%), *Salmonella* Rissen (16.3%), and *Salmonella Mbandaka* (12.6%) [1]. The analysis of antimicrobial resistance revealed that a staggering 80.0% of the isolates exhibited multidrug resistance, encompassing resistance to sulfamethoxazole (84.5%), lincomycin (89.4%), and ampicillin (96.9%), etc. [1]. Moreover, a previous report indicated that most *Salmonella derby* strains have the ability to form a biofilm, and then were categorized as non-wildtype or resistant to tetracycline and streptomycin [22]. Therefore, in addition to drug control and physical prevention and control, it may be a long-term goal to find the resistance genes or pathways against *Salmonella* in host cells. Therefore, in the study, we constructed an in vitro infection model for *Salmonella enterica* in IPEC-J2 cells, with RNA-seq being performed to indicate the pathogenic process, and focused on analyzing the alterations in key genes and pathways associated with the antibacterial process.

Post-weaning diarrhea caused by *Salmonella enterica* continues to present a formidable obstacle within the industry, leading to compromised performance and survival rates among weaned pigs, while simultaneously posing an imminent threat with regard to human foodborne bacterial illnesses [23]. Piglets in a previous study were subjected to challenge with *Salmonella*, which resulted in the remarkable up-regulation of key components of the innate immune system at 2 days post -inoculation, including Toll-like receptor cascades, the phagosome pathway, the cytokine signaling pathway, and the lysosome pathway; however, this heightened response was subsequently attenuated at 7 days post-inoculation [24]. Moreover, the MAPK, NOD-like receptor, TLR, and PPAR signaling pathways, among others, were enriched in chickens after being challenged with *Salmonella Typhimurium* [25]. The study indicated that bile secretion, mineral absorption, and retinol metabolism were up-regulated significantly, while human T-cell leukemia virus 1 infection, the P53 signaling pathway, and the Toll-like receptor signaling pathway were down-regulated significantly. The results may indicate that the rapid immune response phase passed and that the metabolic process within the cell was affected. These results seem to be consistent with the results of Zhou et al.’s study [26], and they suggest that the host response to bacterial infection is not limited to immune-related pathways, but that there is also a response to metabolic pathways in host cells. Meanwhile, it is well known that LPS is usually used to construct a cellular inflammation model in vitro [14,16], and so the transcriptomic profile of IPEC-J2 cells under LPS treatment was characterized. Based on the integrative analysis of the transcriptomic profile of IPEC-J2 cells under *Salmonella enterica* and LPS exposure, 368 up-regulated genes and 101 down-regulated genes were shared, which accounted for the majority of DEGs induced by Salmo *Salmonella enterica* and LPS. Moreover, the top pathways, such as those for bile secretion, mineral absorption, insulin secretion, human papilloma virus 1 infection, Toll-like receptor signaling, and PPAR signaling, were all enriched significantly under *Salmonella enterica* and LPS exposure. Thus, we speculate that the genes’ expression patterns in IPEC-J2 cells after *Salmonella* infection are largely similar to those after LPS treatment in our study.

Among these pathways, that for bile secretion is an important function of the liver that allows the elimination of hepatic metabolites such as bilirubin, cholesterol, drugs, and toxins, as well as facilitating the intestinal absorption of lipids and fat-soluble vitamins [27]. Biliary secretions provide bile acids crucial for the digestion and absorption of fats, facilitate the elimination of metabolites and xenobiotics, and contribute additional bicarbonate ions to ensure optimal buffering in the duodenum [28]. However, the function and mechanism of this pathway in bacterial infection remain relatively unknown. Bile acids contribute to the regulation of inflammation and are associated with intestinal inflammatory diseases, and gut microbes can convert bile acids into immune signaling molecules involved in the regulation of intestinal inflammation [29,30]. Therefore, the results indicate that the bile secretion pathway should be involved in the response to *Salmonella enterica* and LPS exposure in IPEC-J2 cells. Interestingly, among the genes of the bile secretion pathway, AQP8 is specifically expressed in the colon, rectum, and pancreas in humans based on the Human Protein Atlas database (Figure S2A). Moreover, in porcine, AQP8 is specifically expressed in the gastrointestinal system, including the stomach, duodenum, jejunum, and colon (Figure S2B). AQP water channels are one of the main transcellular pathways involved in fast transepithelial fluid transport [31]; the process involves a cell membrane protein that regulates the movement of water into and out of the cell, exerting control over its flow [32]. Previous reports indicated that AQP8 water channels in hepatocyte bile canalicular membranes facilitate water transport during bile secretion [33,34,35]. A knockdown of AQP8 cells resulted in a significant 70% decline in canalicular volume, indicating impaired basal canalicular water movement. Additionally, the reduced expression of AQP8 inhibited canalicular water transport by −65% under an inward osmotic gradient and by −80% when stimulated with the bile secretory agonist dibutyryl cAMP [34]. The defective expression of AQP8 water channels contributes to cholestatic hepatocytes’ dysfunction in bile secretion [35]. Moreover, the regulation of aquaporins propelled by osmotic forces constitutes a prominent pathway for water transportation within the epithelium of the digestive tract [36]. Among these factors, AQP8 exhibits high expression levels in the colon [37]. Therefore, a low expression of AQP3 and AQP8 leads to reduced water reabsorption in the colonic mucosa, causing diarrhea [38]. Moreover, AQP8 was down-regulated in a rat model of diarrhea-predominant irritable bowel syndrome (IBS-D); however, atractylodes oil alleviates IBS-D by regulating intestinal inflammation and the intestinal barrier, while increasing the expression of AQP8 [32]. 

The results indicated that the up-regulation of AQP8 induced by atractylodes oil was involved in modulating intestinal inflammation and the intestinal barrier, yielding promising outcomes. In our study, the mRNA and protein expressions of AQP8 were higher under *Salmonella enterica* infection and LPS treatment. Therefore, we hypothesize that the increase in AQP8 may be a response of the cells to inflammation and injury induced by *Salmonella enterica* and LPS exposure, and that it has a favorable outcome. Thus, AQP8 and the bile secretion pathway will be important molecular targets in a future study.

## 5. Conclusions

In summary, our study fully illustrated the transcriptional landscape of *Salmonella enterica* infection and LPS treatment in IPEC-J2 cells. The results describe the enriched biological processes and signaling pathways, pointing out that a large number of metabolic pathways are enriched; among them, the pathway of bile secretion may be an important pathway in the regulation of the prevention of *Salmonella enterica* infection. Moreover, AQP8 may be used as a resistance gene against *Salmonella enterica* infection, intestinal inflammation, and other related diseases in pigs. In general, our findings provide a theoretical foundation for the identification of molecular markers associated with *Salmonella enterica* infection, and for the implementation of preventive and control strategies in livestock farming.

## Data Availability

The entirety of the data and materials utilized in this study can be made available upon a reasonable request directed towards the corresponding author. The data are not publicly accessible for the time being due to the inclusion of additional topics.

## Supplementary Materials

The following supporting information can be downloaded at https://www.mdpi.com/article/10.3390/ani14050789/s1: Figure S1: Top 20 enriched KEGG pathway terms for the differentially expressed genes of IPEC-J2 under *Salmonella enterica* vs. LPS exposure; Figure S2: Expression of AQP8 in different tissues of humans and pigs; Table S1: Oligonucleotide sequences and size of primers; Table S2: Differentially expressed genes in IPEC-J2 between *Salmonella enterica*-treated and control groups; Table S3: GO term enrichment in up- and down-regulated genes between *Salmonella enterica* infection group and control group; Table S4: Signaling pathway associated with up- and down-regulated genes between *Salmonella enterica* infection group and control group; Table S5: Differentially expressed genes in IPEC-J2 between the LPS-treated and control groups; Table S6: GO term enrichment in up- and down-regulated genes between LPS-treated and control groups; Table S7: Signaling pathway associated with up- and down-regulated genes between LPS-treated and control groups; Table S8: Differentially expressed genes in IPEC-J2 that *Salmonella enterica*-infected and LPS groups have in common; Table S9: GO term enrichment in up- and down-regulated genes that the *Salmonella enterica* infection and LPS groups have in common; Table S10: Signaling pathway associated with up- and down-regulated genes that the *Salmonella enterica* infection and LPS groups have in common; Table S11: Differentially expressed genes in IPEC-J2 between the *Salmonella enterica*-infected and LPS groups; Table S12: GO term enrichment in up- and down-regulated genes between *Salmonella enterica* infection and LPS-treated groups; Table S13: Signaling pathway associated with up- and down-regulated genes between *Salmonella enterica* infection and LPS-treated groups.
