# Supplementary material for: The Metabolic Pathway of Bile Secretion Is Vulnerable to Salmonella enterica Exposure in Porcine Intestinal Epithelial Cells
Source: Animals (Basel). 2024 Mar 3;14(5):789. doi: 10.3390/ani14050789 (PMC10931422; doi:10.3390/ani14050789)
Supplement: Supplementary file 1 [file animals-14-00789-s001.zip › Supplement Figures .pdf]

Transcriptome profiling reveals important regulation genes and pathway signals in IPEC-J2 Cells of pigs mediated by *Salmonella Enterica*

Jiajia Cai, Xiaolei Chen, Haifei Wang, Shenglong Wu, Demei Cai and Hairui Fan

Supplement Figures:

Figure S1

The top 20 enriched KEGG pathway terms for the differentially expressed genes of IPEC-J2 under Salmonella vs LPS.

Figure S2

The expressed of *AQP8* in different tissue of human and pig.

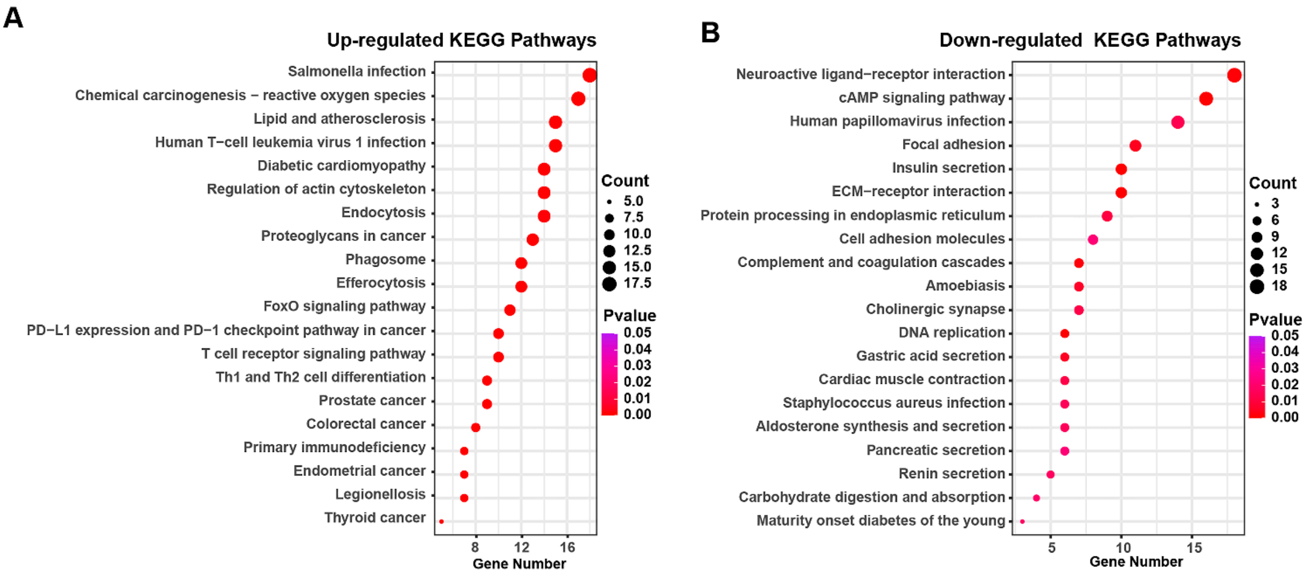

Figure S1. The top 20 enriched KEGG pathway terms of up-regulated genes (A) and down-regulated genes (B) in IPEC-J2 under Salmonella vs LPS.

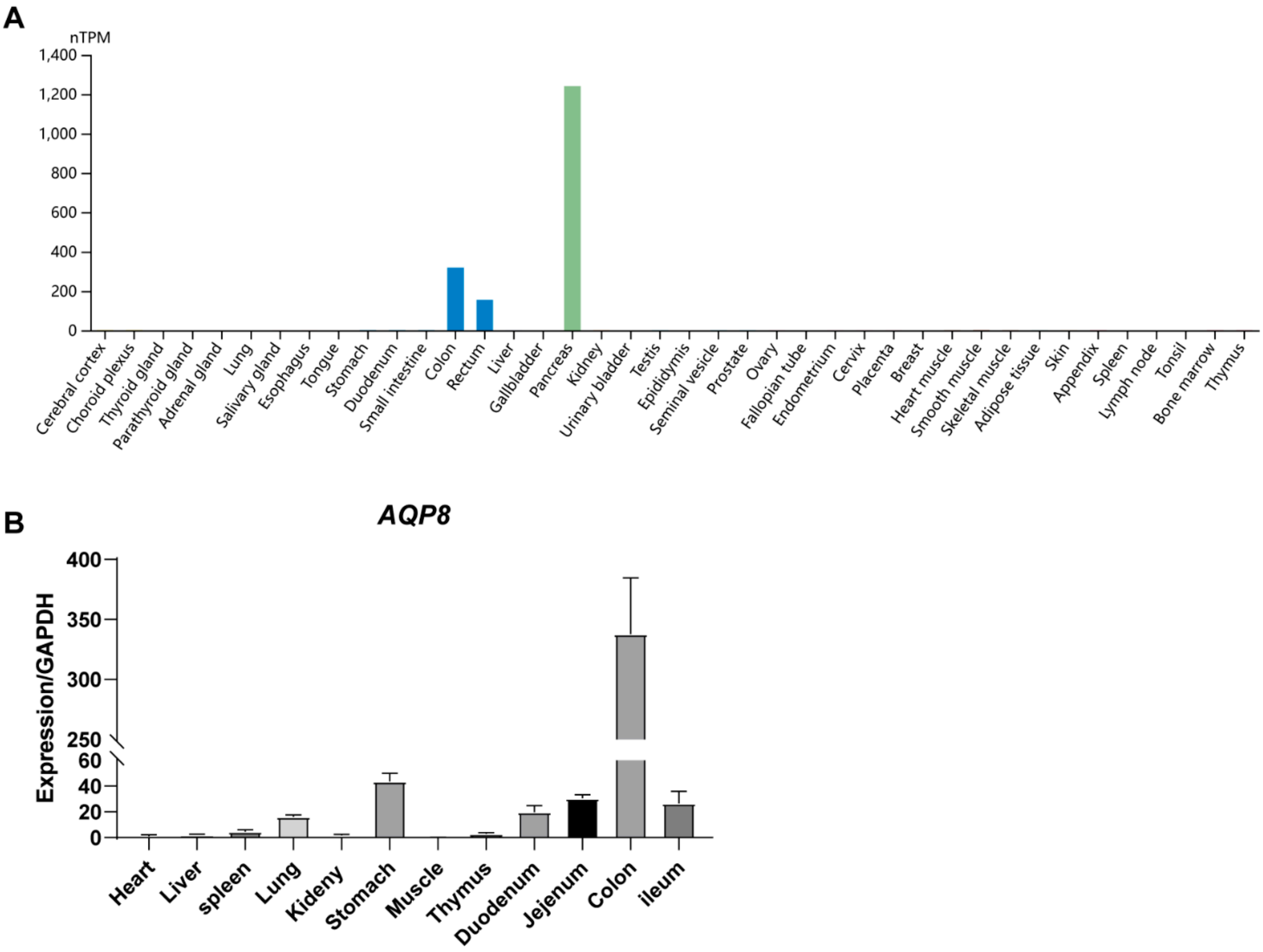

Figure S2

The expression of *AQP8* in different tissue of human and pig. **(A)** the expression of *AQP8* in different tissue of human based on Human Protein Atlas database. **(B)** The mRNA expression of *AQP8* in different tissue of pig by RT-qPCR analysis.
