# Supplementary material for: The Metabolic Pathway of Bile Secretion Is Vulnerable to Salmonella enterica Exposure in Porcine Intestinal Epithelial Cells
Source: Animals (Basel). 2024 Mar 3;14(5):789. doi: 10.3390/ani14050789 (PMC10931422; doi:10.3390/ani14050789)
Supplement: Supplementary file 1 [file animals-14-00789-s001.zip › Table S1 Oligonucleotide sequences and size of primers.pdf]

**Table S1 Oligonucleotide sequences and size of primers**

| GENE | Sequence | Product length (bp) |
|---|---|---|
| *hilA* | F: 5'-TCCACGCAGGAAATAACAGG-3'<br>R: 5'-TGGGCAACCAGCACTAACG-3' | 203 |
| *Inv1* | F: 5'- GGCGATCCTTGAACAAATAGC -3'<br>R: 5'- CGGCGAACAATAGACTGCTT -3' | 145 |
| *16S* | F: 5'- GGCGATCCTTGAACAAATAGC -3'<br>F: 5'- GGCGATCCTTGAACAAATAGC -3' | 180 |
| *TNF-α* | F: 5'-CGACTCAGTGCCGAGATCAA-3'<br>R: 5'-CTCACAGGGCAATGATCCCA-3' | 85 |
| *IL-6* | F: 5'-CTGGGTTCAATCAGGAGACCT-3'<br>R: 5'-TTCCCTTTTGCCTCAGGGTC-3' | 165 |
| *IL-1β* | F: 5'-GGCTACTGCCTTCCCTACC -3'<br>R: 5'-CCTGATTGAACCCAGATTGG-3' | 260 |
| *ABHD2* | F: 5'- GATCAGTGTCGGCGCTTCTA -3'<br>R: 5'- AGCCATGAAACTTCCGCATC -3 | 193 |
| *AQP8* | F: 5'- GAGGTCTCCACCTGACGATG -3'<br>F: 5'- CTCCTCAGGACTCACTGCCTTG -3' | 98 |
| *LRFN3* | F: 5'- ACAGCTGCTCTTGTCCAGTG -3'<br>F: 5'- GCGAGGTCTGTCAACAGGAA -3' | 146 |
| *THEM6* | F: 5'- TCCCTGAATCCACCGCTTAC -3'<br>F: 5'- AGATTACGCCTCCGATGTGG-3' | 203 |
| *DIAPH3* | F: 5'- CACTGACGGATGATGTGCTG -3'<br>F: 5'- GTCCTTTTCCCGCAATGGTG -3' | 236 |
| *KLHL3* | F: 5'- CGAAGAGAATGTCCAGGTGC -3'<br>F: 5'- CGGTCAGCTTGTCACTGGAT-3' | 272 |
| *GAPDH* | F: 5'-ACATCATCCCTGCTTCTACTGG-3'<br>R: 5'-CTCGGACGCCTGCTTCAC-3' | 188 |
